# Metabolic response of glioblastoma cells associated with glucose withdrawal and pyruvate substitution as revealed by GC-MS

**DOI:** 10.1186/s12986-016-0131-9

**Published:** 2016-10-18

**Authors:** Henry Oppermann, Yonghong Ding, Jeevan Sharma, Mandy Berndt Paetz, Jürgen Meixensberger, Frank Gaunitz, Claudia Birkemeyer

**Affiliations:** 1 Klinik und Poliklinik für Neurochirurgie, Universitätsklinikum Leipzig AöR, Liebigstraße 19, Leipzig, 04103 Germany; 2 Institut für Analytische Chemie, Fakultät für Chemie & Mineralogie der Universität Leipzig, Linnéstraße 3, Leipzig, 04103 Germany

**Keywords:** Cancer, Glucose, Pyruvate, Metabolite profiling, Glioblastoma, Warburg effect

## Abstract

**Background:**

Tumor cells are highly dependent on glucose even in the presence of oxygen. This concept called the Warburg effect is a hallmark of cancer and strategies are considered to therapeutically exploit the phenomenon such as ketogenic diets. The success of such strategies is dependent on a profound understanding of tumor cell metabolism. With new techniques it is now possible to thoroughly analyze the metabolic responses to the withdrawal of substrates and their substitution by others. In the present study we used gas chromatography coupled to mass spectrometry (GC-MS) to analyze how glioblastoma brain tumor cells respond metabolically when glucose is withdrawn and substituted by pyruvate.

**Methods:**

Glioblastoma brain tumor cells were cultivated in medium with high (25 mM), medium (11 mM) or low (5.5 mM) glucose concentration or with pyruvate (5 mM). After 24 h GC-MS metabolite profiling was performed.

**Results:**

The abundances of most metabolites were dependent on the supply of glucose in tendency but not in a linear manner indicating saturation at high glucose. Noteworthy, a high level of sorbitol production and release was observed at high concentrations of glucose and high release of alanine, aspartate and citrate were observed when glucose was substituted by pyruvate. Intermediates of the TCA cycle were present under all nutritional conditions and evidence was found that cells may perform gluconeogenesis from pyruvate.

**Conclusions:**

Our experiments reveal a high plasticity of glioblastoma cells to changes in nutritional supply which has to be taken into account in clinical trials in which specific diets are considered for therapy.

**Electronic supplementary material:**

The online version of this article (doi:10.1186/s12986-016-0131-9) contains supplementary material, which is available to authorized users.

## Background

In many studies published in recent years the analysis of tumors predominately focused on gene and protein expression as well as on signal transduction in order to draw conclusions about the biology of cancer. Compared to the high amount of literature focusing on these topics much less has been investigated with regard to cancer cell metabolism despite the fact that the peculiarities of energy metabolism in tumors have already been investigated at the beginning of the last century [1].

The most prominent feature of tumor cell metabolism is the so-called Warburg effect which describes a strong dependence on glycolytic production of ATP accompanied by the conversion of pyruvate to lactate even in the presence of oxygen (aerobic glycolysis). In his article from 1956 which was a translation of a lecture delivered in 1955, Warburg still proposed that “respiration” (OxPhos) must be irreversibly injured in cancer cells [2]. Although the concept has been refined, it is still common sense that in many cancer cell lines as well as in solid tumors the production of lactate is more pronounced than the production of pyruvate. Pyruvate accounts for 60–75 % of the metabolic consumption of external glucose whereas the production of acetyl-CoA from pyruvate accounts for ~10 to 25 % of the glycolytic flux [3, 4]. At this point it should also be kept in mind that only a fraction of the acetyl-CoA produced from pyruvate is used for the production of ATP [3, 4].

Aerobic glycolysis is still considered to be a hallmark of cancer [5] and up to now no breakthrough therapeutic strategy exploiting this potential Achilles heel of cancer has been developed. Aside from research on drugs that could possibly be used for a “metabolic therapy” (for review see [6]), several authors and clinicians focused on the development of diets especially high in fat and low in carbohydrates. These so-called ketogenic diets were considered having beneficial effects by forcing cells to utilize fatty acids as their primary energy source [7–10]. Aside from the increasing amount of data implying that the ketogenic diet is an effective adjuvant cancer therapy (for a summary of current mouse models and clinical trials see [11]), the underlying mechanisms might be more complex involving also anti-angiogenic, anti-inflammatory and pro-apoptotic processes [12]. However, it is without question that the development of successful strategies targeting tumor-specific metabolism requires a thorough understanding of the underlying mechanisms, especially of the mechanisms that enable tumor cells to switch from one substrate to another under defined dietary constraints. Unfortunately, up to now, most investigations focused on proteins required for the glycolytic flux and much research has been committed to the analysis of their activity [6, 13]. In addition, a number of transcription factors and pathways involved in the up-regulation of the corresponding genes have frequently been identified to be aberrantly regulated in cancer such as c-Myc, Hif-1α or mTOR [14]. Much less research has been done on the metabolites and their concentration as the available methods have been cumbersome and mostly out of fashion. With the advent of improved technologies such as liquid or gas chromatography (GC) coupled to mass spectrometry (MS) the biochemical analysis of pathway fluxes and changes of single metabolite concentrations under different physiological conditions became possible in an elegant and precise manner.

In the present work we used GC coupled to MS to analyze the metabolic response of glioblastoma cells to the presence of different concentrations of glucose and in a situation when glucose is substituted by pyruvate. Glioblastoma is the most aggressive and most frequent primary brain tumor in adults with a median survival after biopsy and standard therapy of only 14.6 months [15]. As previous experiments pointed towards the importance of glycolysis for the survival of cells derived from this highly malignant tumor and the possibility that pyruvate may become important under conditions of impaired glycolysis [16], we analyzed metabolite abundances under different concentrations of glucose and in medium in which glucose was substituted by pyruvate. In addition, we omitted medium supplements commonly used such as fetal bovine serum or glutamine which significantly influence metabolic fluxes and in the case of serum in an even undefined manner.

## Methods

### Chemicals and reagents

If not stated otherwise all chemicals were purchased from Sigma-Aldrich (Taufkirchen, Germany).

### Cell culture

U87 cells were originally obtained from the ATCC (Manassas, USA) and cultured in 250 mL culture flasks (Sarstedt AG & Co., Nümbrecht, Germany) using DMEM/25 mM glucose, without pyruvate (Life Technologies, Darmstadt, Germany) supplemented with 10 % fetal bovine serum (FBS superior, Biochrom, Berlin, Germany), 2 mM GlutaMAX and antibiotics (Life Technologies) at 37 °C and 5 % CO_2_ in humidified air in an incubator. In order to confirm identity over long culture periods, cells were genotyped by STR analysis at the Genolytic GmbH (Leipzig, Germany) using a PowerPlex® 21 System (Promega, Mannheim, Germany) and cells were confirmed as the U87MG cell line from the ATCC [17]. For starvation experiments, U87 cells were seeded in 6-well plates (TPP, Trasadigen, Switzerland) at a density of 10^6^ cells per well in 2 mL full supplemented DMEM (10 % FBS, GlutaMAX, antibiotics) and incubated for 3 h before receiving fresh medium (1 mL) without a carbon source and without GlutaMAX and FBS (DMEM [0]). Cells were incubated for 20 h before replacing the culture medium with medium (1 mL) containing either different concentrations of glucose or medium without glucose but pyruvate at a concentration of 5 mM.

### Determination of extracellular lactate

After incubation, media were collected for lactate determination and cells were lysed in 200 μL lysis buffer (77 mM K_2_HPO_4_, 23 mM KH_2_PO_4_, 0.2 % TritonX-100, pH 7.8) and the protein content was determined using the Pierce 660 nm Protein Assay (Thermo Scientific, Braunschweig, Germany). For D-lactate determination collected medium (100 μL) was evaporated to dryness and dissolved in H_2_O_dd_ (25 μL). For the determination of L-lactate, H_2_O_dd_ (20 μL) was added to collected medium (5 μL). Then, prepared samples (20 μL) were incubated in the wells of a black 96 well plate (Greiner Bio One, Frickenhausen, Germany) in the presence of enzyme mix (224 μL) consisting of 430 mM glycine, 340 mM hydrazine sulfate, 5 mM NAD and 10 U L-lactate dehydrogenase (Megazyme, Wicklow, Ireland) (pH 9) for L-lactate and 490 mM glycine, 200 mM hydrazine sulfate, 1 mM DETAPAC, 5 mM NAD and 100 U D-lactate dehydrogenase (pH 9.2) for D-lactate (Megazyme) (final concentration for all formulations) for 90 min at room temperature. Production of NADH was observed by fluorometric detection (excitation/ emission = 340/ 460 nm) using a SpectraMax M5 Microplate Reader (Molecular Devices, Biberach, Germany) [18]. A standard curve was prepared using 0.375 to 10 μg D/L-lactate.

### Determination of free MGO

U87 cells were seeded as described above. After 20 h in medium without a carbon source and without serum, cells received fresh medium (1 mL) supplemented with the compounds to be tested. After 24 h of incubation, cells were washed with ice cold washing buffer (1 mL of 100 mM Tris/HCl, pH 8.0) and then 70/30 methanol/H_2_O (*v/v*; 430 μL) containing 1.16 mM *O*-(2,3,4,5,6-pentafluorobenzyl)hydroxylamine (PFBHA) was instantly added to each well. After shaking for 10 min at room temperature, the plates were placed on a shaking incubator at 40 °C for 1 h derivatization. Afterwards, the solution was transferred into a 1.5 mL reaction vial. Then, 9 M H_2_SO_4_ (10 μL) and cyclohexane (200 μL) was added to each sample. The solution was vortex mixed, followed by a brief centrifugation. The upper organic phase was transferred into a clean conical glass insert. This extraction procedure was performed three times in total. The collected extract was evaporated to dryness using a gentle stream of air, analytes were dissolved in cyclohexane (60 μL) containing suberic acid dimethyl ester as internal standard (cyclohexane: internal standard = 10000:1, *v/v*) and analyzed by GC-MS.

### GC-MS analysis of intracellular MGO samples

Free MGO from cell extracts was analyzed on an Agilent 6890 N gas chromatograph equipped with a 7683 Series auto sampler and a 30-m J&W Fisher DB-35 ms capillary column (250-μm I.D. and 0.25 μm film) coupled with a 5973 N mass selective detector (all modules and columns from Agilent Technologies, Waldbronn, Germany). Samples (2 μL) were injected at 250 °C in splitless mode with helium as carrier gas with a flow rate of 1 mL/min. Initial GC oven temperature was set to 50 °C, held for 2 min, then increased at a rate of 15 °C/ min up to 320 °C and held for 10 min. The electron impact ionization source operated at 230 °C, 70 eV and a scan range of *m/z* 50 to 550. For MGO quantitation, the peak area of *m/z* 181 of the corresponding derivative was integrated.

### Metabolic profiling via GC-MS

For the determination of extracellular metabolites, medium (10 μL) was collected from each well and immediately frozen at –80 °C until further use. For the determination of intracellular metabolites, cells were briefly washed with pre-cooled (4 °C) washing buffer on ice. Immediately after washing, pre-cooled (-20 °C) methanol (1 mL) was added to each well and metabolites were extracted for 24 h on an orbital shaker at 8 °C. Then, the extracts were transferred to 1.5 mL reaction vials and additional pre-cooled (4 °C) methanol (500 μL) was used to rinse the remaining metabolites from each well and combined with the extract. Samples were evaporated to dryness using a speed vac (Maxi-Dry Lyo, Heto-Holten, Allerød, Denmark) and stored at -80 °C until further use.

Derivatization and GC-MS analyses were performed as described previously [19]. Data evaluation was carried out using AMDIS 2.71 [20] for peak picking and creation of a customer library of detected peaks. Quantitation with Xcalibur 1.4 (Thermo Scientific) was based on the integration of selective mass traces. Tentative identifications were achieved by spectra comparison with NIST14 (National Institute of Standards and Technologies [NIST], Gaithersburg, USA) and a customer library of reference spectra under consideration of related Kovac retention time indices [21]. Metabolite profiling experiments were repeated once with a similar result, whereas representative data is presented. If not stated otherwise, the abundance of a metabolite is defined by the peak area determined from the selected ion chromatogram of an experiment normalized to total cellular protein (μg).

### Statistical analysis

Student’s *t*-test was performed using the algorithm implemented in Excel (Version: 14.0.7128.5000; Microsoft, Redmond, USA) (unpaired two-sample test with unequal variances). Principal component analysis was performed using the Excel add-in Multibase package (Numerical Dynamics, Japan). All experiments were carried out in 6-tuplicate.

## Results

### D-lactate, L-lactate and MGO production at different concentrations of glucose and supply of pyruvate

In order to investigate how different concentrations of glucose in the medium contribute to the glycolytic flux in U87 glioblastoma cells, we determined the production of L-lactate in medium with different concentrations of glucose and in the presence of 5 mM pyruvate instead of glucose. In addition, we also determined the production of methylglyoxal (MGO) and D-lactate. MGO arises by non-enzymatic elimination of phosphate from glyceraldehyde-3-phosphate and dihydroxyacetone phosphate, two intermediates of glycolysis, and is finally converted to D-lactate by the glyoxalase system [22]. We expected to get a more comprehensive picture of the glycolytic flux than just by the determination of L-lactate, which only appears as long as glycolytic produced pyruvate is not used for the production of acetyl-CoA. For the experiment, cells were cultivated for 20 h in the absence of glucose and pyruvate in medium which did not contain any glutamine source or fetal bovine serum. Then, fresh medium was added containing 25, 11 and 5.5 mM glucose or 5 mM pyruvate without glucose, followed by incubation for 24 h. The used concentrations of glucose are commonly employed in cell culture experiments. With regard to physiological concentrations, 5 mM has to be considered as a physiological blood concentration at starvation and 11 mM as the blood concentration after a meal, whereas 25 mM is only reached in diabetic conditions. Blood concentrations of pyruvate are supposed to be around 0.05 mM (0.44 mg/100 ml) in fasted individuals [23]. The rational to use an almost 100-fold higher concentration is based on control experiments in which we determined that at this concentration (5 mM) the relative ATP concentration is saturated and was comparable to that obtained with all three concentrations of glucose used (Additional file 1: Figure S1). Finally, media were collected for the determination of extracellular D- and L-lactate. In addition, the intracellular MGO was determined from the cells and normalized to the total extracted protein.

**Fig. 1:**
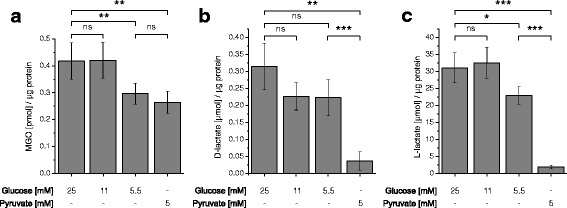
D-Lactate, L-lactate and MGO production at different concentrations of glucose and in medium containing pyruvate. U87 cells were cultivated in the presence of 5.5 mM, 11 mM, 25 mM glucose or 5 mM pyruvate in the absence of glucose. After 24 h intracellular MGO (**a**) as well as extracellular D-lactate (**b**) and L-lactate (**c**) were determined. All experiments have been conducted in 6-tuplicate and statistical significance was determined by Student’s *t*-test with: *: *p* < 0.05; **: *p* < 0.005; ***: *p* < 0.0005; ns: not significant

As can be seen in Fig. 1, there was no significant difference neither in L/D-lactate production nor in MGO production between cells either incubated in the presence of 25 mM or 11 mM glucose. This may indicate that there was almost the same glycolytic flux at both concentrations of glucose. Only when the concentration of glucose was further decreased down to 5.5 mM, a significant reduction was detected in the production of L-lactate (fold change to: 0.74 ± 0.08; *p* < 0.05) which was very low in the absence of glucose and the presence of pyruvate (fold change to: 0.06 ± 0.02; *p* < 0.0005). Although a significant reduction of MGO was observed at a concentration of 5.5 mM glucose compared to higher glucose concentrations (fold change in 5.5 compared to 25 mM glucose: 0.71 ± 0.09; *p* < 0.005) the abundance of MGO was almost equal to the one measured in the absence of glucose (fold change in 5 mM pyruvate compared to 25 mM glucose: 0.63 ± 0.09; *p* < 0.005) indicating that the highly reactive MGO is a poor indicator of the glycolytic flux. The same holds true for D-lactate although compared to MGO it was strongly reduced in the absence of glucose (fold change in 5 mM pyruvate compared to 25 mM glucose: 0.12 ± 0.09; *p* < 0.005).

### Metabolites in glioblastoma cells at different supply with glucose and pyruvate

**Fig. 2:**
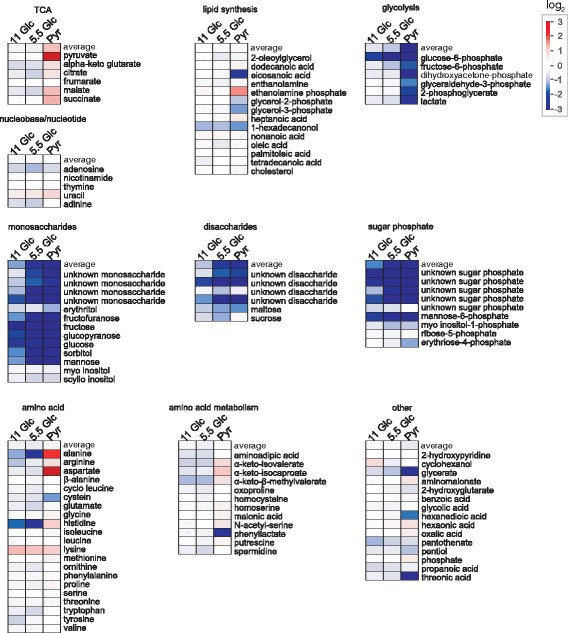
Comparative metabolic profiling of U87 cells cultivated in the presence of glucose and pyruvate. Fold changes (log_2_-transformed) of intracellular metabolite abundances are indicated with regard to the highest concentration of glucose employed (25 mM; set to log_2_(1) = 0) for glucose concentrations of 11 mM (11 Glc), 5.5 mM (5.5 Glc) or for medium without glucose containing 5 mM pyruvate instead (Pyr). Identified metabolites were grouped in dependence on their metabolic role: TCA: metabolites associated with the tricarboxylic acid cycle; lipid synthesis: metabolites associated with lipid synthesis; glycolysis: glycolytic metabolites; nucleobase/nucleotides; monosaccharides; disaccharides; sugar phosphates; amino acids; amino acid metabolism: metabolites associated with amino acid metabolism; other: other metabolites not unequivocally associated with the other pathways indicated. The average of the fold changes from the metabolites in each group is indicated as “average”

In order to analyze how metabolites are affected by a different supply of glucose we analyzed the associated metabolic changes using GC-MS profiling. Therefore, U87 cells were cultivated as described in the previous section and after 24 h of incubation the intra-and extracellular metabolites were extracted, derivatized and analyzed by GC-MS. After automated peak deconvolution by AMDIS, 194 peaks were manually selected and quantified using Xcalibur. Finally, 106 metabolites were identified by comparison of mass spectra and Kovac retention time indices with mass spectral libraries. The resulting relative intracellular abundances are presented in Fig. 2 as logarithmically transformed fold-changes compared to the samples obtained from the cells treated with 25 mM glucose.

**Fig. 3:**
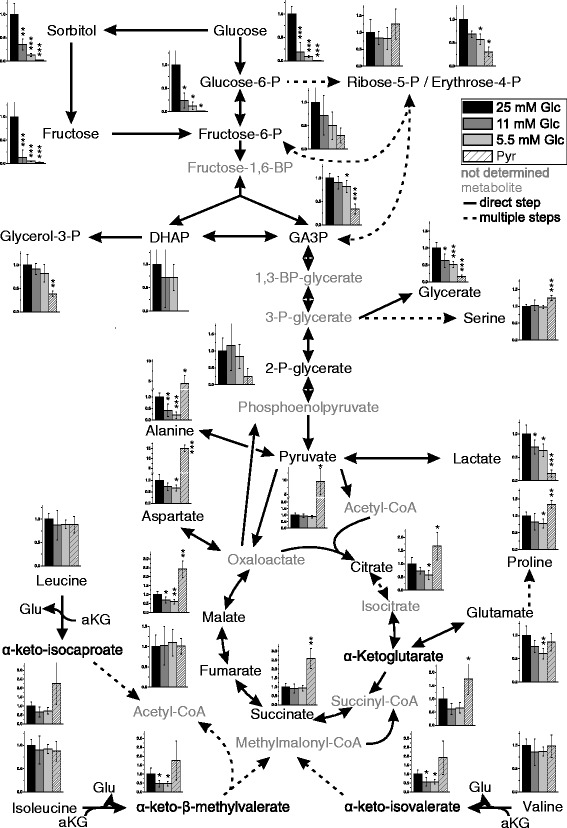
Metabolic pathway of U87 cells profiling data. A combined metabolic pathway is presented, including glycolysis, the pentose-phosphate pathway, the TCA cycle and branched chain amino acid degradation. Intracellular metabolite abundances (Fig. 2) are shown as bar diagrams depicting the relative abundance which is the: ratio between the abundance of a metabolite (the peak area) normalized to total protein (μg) compared to the abundance at 25 mM glucose (normalized to protein) set as 1. Direct reactions without intermediates are presented as straight lines and reactions involving several steps are presented as dotted lines. Metabolites which were not determined are labeled in Grey. All experiments have been conducted in 6-tuplicate and statistical significance was determined by Student’s *t*-test with: *: *p* < 0.05; **: *p* < 0.005; ***: *p* < 0.0005. An extended version of the figure including extracellular metabolite concentrations is available in the (Additional file 2: Figure S2). Abbreviations: GA3P: glyceraldehyde-3-phosphate; DHAP: dihydroxyacetone phosphate; Glu: glutamate; aKG: α-ketoglutarate

Among the most prominent changes associated with a reduced supply of glucose was an expected reduction in the amount of different mono- and disaccharides and sugar phosphates that already became prominent when the concentration of glucose was reduced from 25 mM to 11 mM. In contrast to the results obtained with the analysis of MGO and lactate in the previous section, the metabolic profile of 11 mM glucose samples was more similar to 5.5 mM glucose than to 25 mM glucose samples, which was confirmed by multivariate analysis (Additional file 2: Figure S2). Possibly, this may be related to the fact that the glycolytic flux appeared to enter saturation from 11 mM glucose supplement on. A complete overview of the metabolites detected and quantified and their relation to other metabolites is depicted in Fig. 3 (for an extended version see Additional file 3: Figure S3). Based on the data presented in Fig. 3, a number of different features of tumor metabolism in U87 cells under different nutritional supply was analyzed and is presented in the following paragraphs.

### Glucose and the glycolytic flux

As can be seen in Fig. 3 the intracellular abundance of glucose increases with increased supply, but does not follow it in a linear manner as the intracellular abundance of glucose was more than 5 times higher at an extracellular concentration of 25 mM than at 11 mM. We presume that the transport of glucose is mainly carried out by GLUT1 as this is the main transporter in brain [24] and we found its mRNA to be ~ 25 times more abundantly expressed than that encoding GLUT3 or GLUT4 whereas mRNA encoding GLUT2 was almost not present in the U87 cells used in the experiment (Additional file 4: Figure S4a). As the K_m_ of GLUT1 for glucose is around 1.5 mM, uptake should already be saturated at the lowest concentration of glucose (5.5 mM). Consequently, phosphorylation to glucose-6-phosphate becomes important for a continuous uptake of glucose. This phosphorylation should be catalyzed by hexokinase 2 and hexokinase 1 which at the level of the mRNA is three times stronger expressed than isoform 2 as revealed by qRT-PCR (Additional file 4: Figure S4b). At this point, it is interesting to note, that in normal brain tissue hexokinase 2 is only negligibly expressed and its expression in glioblastoma was already considered to contribute to the Warburg effect and the malignity of the tumor [25].

When glucose in the medium was reduced from 25 mM to 11 mM the abundances of intracellular glucose (fold change to: 0.22 ± 0.19; *p* < 0.0005) and glucose-6-phosphate (fold change to: 0.24 ± 0.16; *p* < 0.05) were significantly reduced. In the case of fructose-6-phosphate (fold change to: 0.72 ± 0.42) and glyceraldehyde-3-phosphate (fold change to: 0.9 ± 0.13) only weak and non-significant effects were observed. A further reduction of glucose supply to 5.5 mM did also not significantly reduce fructose-6-phosphate (fold change to: 0.5 ± 0.29) and 2-phospho-glycerate (fold change to: 0.84 ± 0.35), but we observed a significant reduction in intracellular glucose (fold change to: 0.15 ± 0.02; *p* < 0.0005), glucose-6-phosphate (fold change to: 0.12 ± 0.08; *p* < 0.05) and glyceraldehyde-3-phosphate (fold change to: 0.82 ± 0.13; *p* < 0.05). Comparing glucose concentrations 11 mM and 5.5 mM in medium, no significant changes of the aforementioned metabolites were detected. Therefore, it has to be assumed that the glycolytic flux downstream from glucose-6-phosphate is similar under the different external glucose concentrations. As the amount of ribose-5-phosphate was also not significantly affected by different concentrations of extracellular glucose it may be assumed that the pentose-phosphate pathway has the same activity at all concentrations of glucose, although we observed a significant reduction of erythrose-4-phosphate comparing its amounts at 25 mM glucose and 5.5 mM (fold change to: 0.57 ± 0.11; *p* < 0.05).

### Fructose and sorbitol

Aside from a steep raise of the intracellular abundances of glucose and glucose-6-phosphate when the extracellular concentration of glucose was increased from 11 mM to 25 mM, we also observed a significant raise in the abundances of intracellular sorbitol (fold change to: 2.86 ± 0.98; *p* < 0.005) and fructose (fold change to: 7.69 ± 9.46; *p* < 0.005). Moreover, raising glucose concentrations from 5.5 mM to 11 mM in medium also resulted in a significant enhancement of sorbitol abundances (fold change to: 2.69 ± 0.62; *p* < 0.05). Interestingly, sorbitol produced by the cells was significantly released into the extracellular medium whereas the extracellular abundance of fructose did not follow its enhanced intracellular production (Additional file 3: Figure S3).

### Glucose and the tricarboxylic acid cycle

The data presented in Fig. 3 shows that the abundance of intracellular lactate (fold change in 5.5 mM compared to 25 mM glucose: 0.65 ± 0.13; *p* < 0.05) followed in tendency the abundance of glyceraldehyde-3-phosphate (fold change in 5.5 compared to 25 mM glucose: 0.82 ± 0.13; *p* < 0.05) which is the metabolite at the entry of the second (three carbon) part of the glycolytic pathway. Although this appears to be in accordance with the Warburg effect, we also observed a significant reduction (fold change) in 5.5 mM glucose compared to 25 mM glucose in the amounts of citrate (0.57 ± 0.17; *p* < 0.05), malate 0.61 ± 0.12; *p* < 0.005) and α-ketoglutarate (0.71 ± 0.18). This clearly indicates that at a high glucose concentration (25 mM), glucose-derived pyruvate is metabolized by the TCA cycle.

### Metabolic flux under substitution of glucose by pyruvate: lactate and metabolites associated with the TCA cycle

When glucose was substituted by pyruvate a strong increase of the intracellular amount of pyruvate was observed (5 mM pyruvate compared to 25 mM glucose: 9.85 ± 4.54; *p* < 0.05) which clearly indicates that the compound is readily taken up by the tumor cells. The high intracellular amount of pyruvate was not accompanied by a corresponding raise of intracellular lactate. As the formation of lactate requires NADH + H^+^, that in the presence of a glycolytic flux is produced by the conversion of glyceraldehyde-3-phosphate to glycerate 1,3-bisphosphate, a lack of reduced NADH may be the reason for the non-appearance of increased lactate production in the absence of glucose. We also observed a significantly higher abundance of the TCA cycle metabolites citrate (1.67 ± 0.52; *p* < 0.05), α-ketoglutarate (1.76 ± 0.59; *p* < 0.05), succinate (2.60 ± 0.56; *p* < 0.005) and malate (2.42 ± 0.46; *p* < 0.005) following the higher abundance of intracellular pyruvate when glucose was substituted by pyruvate (fold change in cells exposed to 5 mM pyruvate compared to cells exposed to 25 mM glucose). Therefore, pyruvate in the absence of glucose can obviously be metabolized by the TCA cycle and it has to be expected that at least two mitochondrial NADH + H^+^ producing steps (from isocitrate to α-ketoglutarate and from α-ketoglutarate to succinyl-CoA) are active. Provided that we found no evidence of enhanced production of lactate in the absence of glucose and high levels of intracellular pyruvate, these reduction equivalents are most likely used for the production of ATP by OxPhos rather than shuttled out of the mitochondria. An intensive use of pyruvate for the production of ATP under the extracellular concentrations employed (5 mM) is also in accordance to the observation that at this concentration ATP production was comparable to the production in medium containing glucose (Additional file 1: Figure S1).

We also observed a significantly higher abundance of extracellular citrate when glucose was substituted by pyruvate (5 mM pyruvate compared to 25 mM glucose: 2.01 ± 0.33; *p* < 0.05). Most likely this citrate originates in the mitochondria by the reaction of acetyl-CoA with oxaloacetate. Under normal conditions this reaction precedes the export of citrate which is then cleaved by cytosolic ATP/citric acid lyase in order to provide acetyl-CoA for lipid synthesis by the cytosolic lipid acid synthase. It may be presumed that in the absence of glucose not enough NADPH for lipid synthesis is available which can be gained from the pentose-phosphate pathway or from the conversion of malate to pyruvate by malic enzyme [EC 1.1.1.40] [26]. This notion is in contrast to the fact that the amount of ribose-5-phosphate was still high in the absence of glucose and the presence of pyruvate. On the other hand, the high abundance of malate in the presence of high concentrations of pyruvate may indicate that its conversion to pyruvate by malic enzyme and the associated production of NADPH is impaired.

### Metabolic flux under substitution of glucose by pyruvate: gluconeogenesis

As pointed out in the introduction several metabolites from the glycolytic pathway are building blocks required for growth and proliferation of tumor cells. Therefore, the question was whether a substitution of glucose by pyruvate contributes to gluconeogenesis sufficient for their supply. As demonstrated in Fig. 3, abundances of 2-phosphoglycerate (fold change to: 0.24 ± 0.24) and fructose-6-phosphate (fold change to: 0.29 ± 0.16) were obviously reduced, whereas reduction of abundances in glyceraldehyde-3-phosphate (fold change to: 0.34 ± 0.1; *p* < 0.0005), glycerol-3-phosphate (fold change to: 0.38 ± 0.06; *p* < 0.005) and erythrose-4-phosphate (fold change to: 0.31 ± 0.1; *p* < 0.05) was detected as statistically significant when glucose was substituted by pyruvate (comparing cells exposed to 5 mM pyruvate to cells exposed to 25 mM glucose). Interestingly, the abundance of ribose-5-phosphate did not appear to be affected in a comparable manner as the aforementioned metabolites of the glycolytic pathway when glucose was substituted by pyruvate. This may indicate that the gluconeogenic pathway is active in the absence of glucose and the presence of pyruvate [27].

### Metabolic flux under substitution of glucose by pyruvate: amino acids

Substituting glucose with pyruvate resulted in a strong increase of alanine (significant fold change to: 4.33 ± 2.01; *p* < 0.05) which is most likely catalyzed by the transamination of pyruvate supplied in high amounts in the medium. A second prominent effect was a strong raise in the concentration of aspartate (significant fold change to: 15.09 ± 1.62; *p* < 0.0005). As aspartate is mainly synthesized by a transamination reaction from oxaloacetate it has to be assumed that under our experimental conditions more oxaloacetate was produced by pyruvate carboxylase than is needed for anaplerosis and gluconeogenesis. As both amino acids are released in significantly higher amounts into the medium (alanine: fold change to: 3.51 ± 0.32; *p* < 0.0005; aspartate fold change to: 9.6 ± 5.63; *p* < 0.05), the cells seem to continuously loose amino groups. Although speculative, we presume that the observed increase in the amounts of branched chain keto acids (BCKA) such as α-keto-isocaproate (4-Methyl-2-oxopentanoate) (fold change compared to 25 mM glucose intracellular: 2.27 ± 1.19; extracellular: 2.18 ± 0.13; *p* < 0.0005), α-keto-β-methylvalerate ((S)-3-Methyl-2-oxopentanoate) (fold change compared to 25 mM glucose intracellular: 1.76 ± 1.09; extracellular: 1.53 ± 0.1; *p* < 0.0005) and of α-keto-isovalerate (3-Methyl-2-oxobutanoate) (fold change compared to 25 mM glucose intracellular: 2.07 ± 1.13; extracellular: 1.69 ± 0.09; *p* < 0.0005) may indicate that the amino groups originate from transamination reactions catalyzed by branched-chain amino acid (BCAA) aminotransferase [EC:2.6.1.42] using L-leucine, L-isoleucine and L-valine as substrates (Additional file 3: Figure S3). This notion is supported by the observation that BCAAs are a major source of amino groups for transamination reactions in the brain [28].

## Discussion

The central aim of this study was to obtain a more comprehensive understanding of the metabolic contribution of glucose and pyruvate in tumor cells. In accordance with the concept of Krall and Christofk [21], who pointed out that metabolic mechanisms that are imperative for tumor growth may be uncovered by subjecting tumor cells to nutrient limitations [29], we used different concentrations of glucose in our experiments and we also substituted glucose by pyruvate. The rational to use pyruvate to replace glucose goes back to the observation that cancer cells proliferate rapidly in the presence of exogenous pyruvate [30]. It was also demonstrated that exogenous pyruvate is required to sustain proliferation of both cancer and non-cancer cells that cannot utilize oxygen [31]. In this work the authors also propose that exogenous pyruvate may be released by tumor adjacent cells. Although, to our knowledge there is no experimental confirmation that non-tumor cells can release pyruvate into the tumor microenvironment, the high expression of monocarboxylate transporters (which transport lactate and pyruvate) in many tumors has led to the assumption that tumor cells may use these transporters for the uptake of metabolically important molecules [30].

Analyzing the dependence of the abundance of glycolytic intermediates from glucose we found no strict linear correlation between the extracellular supply of glucose and the abundance of glycolytic intermediates, but there was a general tendency that most metabolites of the glycolytic and associated pathways are more abundant at a higher concentration of glucose than at a lower concentration.

An interesting observation is the high increase in the abundance of sorbitol in the medium.

The polar character of sorbitol usually should prevent its release by diffusion, which in the case of diabetes results in the so-called diabetic cataract [32]. As intracellular sorbitol does induce osmotic stress [33] it is tempting to speculate whether tumor cells may have acquired mechanisms for the effective removal of sorbitol such as the sorbitol permease described to be present in the rabbit papillary epithelial cell line PAP-HT25 [34]. In conclusion, we consider that the high level of sorbitol produced under conditions of high glucose supply and especially the ability to release this metabolite should be studied in more detail as it may be a possible unique target for therapeutic intervention.

Our data clearly demonstrates that at a glucose concentration of 25 mM glycolytically derived pyruvate is metabolized by the TCA cycle. This may at a first glace appear to be in contrast to the Warburg effect but is in agreement with its present day understanding. As pointed out by DeBerardinis et al. [35], the reduction of substrate oxidation by glioblastoma and other tumor cells can simply be secondary to the metabolic activities needed for biosynthesis rather than being an impairment of oxidative metabolism. At this point it has to be taken into account that the TCA cycle also delivers acetyl-CoA which is required for lipid biosynthesis. In fact, using the paediatric glioma cell line SF188 DeBerardinis et al. [35] could demonstrate that 10 % of total glucose metabolism is utilized for the biosynthesis of fatty acids and nucleotides as well as for other processes such as glycosylation.

In conclusion, our data demonstrates that in human U87 glioblastoma cells, pyruvate derived from glucose can be shuttled into the TCA cycle as previously demonstrated for paediatric glioma cells [35], A549 lung carcinoma cells [4] and rat C6 glioma cells [3]. From our data we cannot estimate the percentage of glucose entering the TCA cycle as we did not use isotopically labeled glucose. This will be the next step in future experiments in which we will also investigate how other energy-rich metabolites such as ketone bodies can be utilized as substrates for glioma cell metabolism.

More experiments are required to solve the interesting question whether the high extracellular concentration of citrate, under the withdrawal of glucose and its substitution by pyruvate, indicates that the cell produces more citrate than needed for the production of fatty acids or whether the conversion of citrate to acetyl-CoA and oxaloacetate is impaired as this would finally prevent tumor cell proliferation. The high amount of citrate released into the medium also raises a question about anaplerosis: it should be kept in mind that the removal of citrate and other metabolites from the TCA cycle that are used as precursors for biosynthetic reactions outside the mitochondria, requires anaplerotic reactions that refill the pool of precursor molecules. In glioblastoma and other transformed cell lines, glutamine is the preferred anaplerotic precursor when it is supplied by the medium, contributing up to 90 % to the oxaloacetate pool [35]. As in our experiments glutamine is not supplied with the medium, the synthesis of oxaloacetate via pyruvate carboxylase becomes important and may compensate the loss of glutamine-dependent anaplerosis, which is a mechanism previously proposed by Cheng et al. who investigated glioblastoma growth under deprivation of glutamine [36]. A high flux of pyruvate through the pyruvate carboxylase reaction is also in agreement with the assumption that the cells perform gluconeogenesis when glucose is substituted by pyruvate in order to provide ribose-5-phosphate (or other metabolites of the glycolytic pathway). At this point it is also interesting to note that gluconeogenesis in tumor cells under low glucose conditions has also been reported in lung cancer cells [27] although in this study glutamine served as substrate for the gluconeogenic pathway.

## Conclusions

Overall the data in the present study demonstrates that i) there is no strict correlation between the extracellular supply of glucose and abundance of glycolytic intermediates, ii) sorbitol is highly produced and released into the medium under high glucose supply, iii) U87 cells metabolize a significant amount of glucose derived pyruvate in the mitochondria and iv) gluconeogenesis is most likely activated when glucose is substituted by pyruvate. These observations suggest a high plasticity of glioblastoma cells to changes in nutritional supply which should be taken into account with regard to the prescription of special diets for the treatment of tumor patients or when considering to use specific inhibitors of glycolysis.

## Additional files

Additional file 1: Figure S1. Relative intracellular ATP concentration at different concentrations of glucose and pyruvate. U87 cells were seeded at a density of 5000 cells per well in 96 well microplates and received medium without a carbon source and without GlutaMAX and FBS for 20 h. Then, fresh medium was added containing different concentrations of glucose (5.5 mM, 11 mM and 25 mM), pyruvate (1 mM, 2.5 mM, 5 mM, 10 mM and 20 mM) or without any carbon source (0 mM). 24 h later the relative intracellular ATP concentration was determined by the CellTiterGlo Assay (for method description see Additional file 5: Methods). Results are represented as mean and standard deviation of 6 independently measured wells compared to the signal of 25 mM glucose set to 1. Statistical significance was determined by Student’s *t*-test with: *: *p* < 0.05; **: *p* < 0.005; ***: *p* < 0.0005; ns: not significant. (TIF 949 kb)
Additional file 2: Figure S2. Multivariate analysis of metabolite profiles. Principal component analysis (PCA) of metabolite profiles from U87 cells cultivated in the presence of 5.5 mM (5.5 Glc), 11 mM (11 Glc), 25 mM glucose (25 Glc) or 5 mM pyruvate in the absence of glucose (Pyr) for 24 h. 25 Glc and Pyr appear clearly separated from each other, whereas 11 Glc and 5.5 Glc have a similar profile separated from 25 Glc and Pyr. PC1 includes 34.7 % of variability (responsible for separation of Pyr), PC2 16.2 % (differences between the glucose treated cells). *n* = 6 for each condition, all identified metabolites were included (*n* = 106). (TIF 69 kb)
Additional file 3: Figure S3. Extended metabolic pathway of U87 cells profiling data. A combined metabolic pathway is presented, including glycolysis, the pentose-phosphate pathway, the TCA cycle and the branched chain amino acid degradation with the subcellular localization (mitochondrial, cytoplasm and extra cellular—methodically it can only be distinguished between intra- and extracellular metabolites). Intracellular metabolite abundances (Fig. 2) are shown as bar diagrams depicting the ratio between the abundance of a metabolite (the peak area) normalized to total protein (μg) compared to the abundance at 25 mM glucose (normalized to protein) set to 1. The consumption of extracellularly present metabolites was determined by the comparison of signals obtained from medium without cells to those after incubation with cells. Thus, a value of 0 indicates the same abundance of a metabolite in medium with and without cells (no consumption) and a value of -1 indicates that the metabolite is completely consumed. Abundances of metabolites released from the cells are expressed as the abundance of a metabolite in medium after incubation compared to its abundance in medium with 25 mM glucose set to 1. All experiments have been conducted in 6-tuplicate and statistical significance was determined by Student’s *t*-test with: *: *p* < 0.05; **: *p* < 0.005; ***: *p* < 0.0005. The statistical analysis for intracellular and released metabolites was performed by comparing the abundance of a metabolite in an experiment (11 mM or 5 mM glucose or 5 mM pyruvate) to the abundance in 25 mM glucose. For consumed metabolites, each metabolite’s abundance was compared to its abundance in medium without cells. Direct reactions are presented as straight lines and reactions involving several steps are presented as dotted lines. Metabolites which were not determined are labeled Grey. Abbreviations: GA3P: glyceraldehyde-3-phosphate; DHAP: dihydroxyacetone phosphate; Glu: glutamate; aKG: α-ketoglutarate; PEP: phosphoenolpyruvate. (JPG 3880 kb)
Additional file 4: Figure S4. mRNA expression of glycolytic genes in U87 cells. Expression of mRNA encoded by the genes GLUT1/2/3/4 (glucose transporters 1/2/3/4) (a) and HK1/2 (hexokinase 1/2) (b) in U87 cells as revealed by qRT-PCR (for method description see Additional file 5). The relative expression was determined using standard curves normalized to the expression of TATA box binding protein (TBP). (TIF 655 kb)
Additional file 5: Methods. (DOCX 16 kb)

## Abbreviations

aKG: α-ketoglutarate
BCAA: Branched-chain amino acid
BCKA: Branched chain keto acids
DHAP: Dihydroxyacetone phosphate
GA3P: Glyceraldehyde-3-phosphate
GC-MS: Gas chromatography coupled to mass spectrometry
Glc: Glucose
Glu: Glutamate
MGO: Methylglyoxal
NIST: National Institute of Standards and Technologies
PCA: Principal component analysis
PEP: Phosphoenolpyruvate
PFBHA: *O*-(2,3,4,5,6-pentafluorobenzyl)hydroxylamine
Pyr: Pyruvate
TCA: Tricarboxylic acid
