# Supplementary figures and images for: Metabolic response of glioblastoma cells associated with glucose withdrawal and pyruvate substitution as revealed by GC-MS
Source: Nutr Metab (Lond). 2016 Oct 18;13:70. doi: 10.1186/s12986-016-0131-9 (PMC5070012; doi:10.1186/s12986-016-0131-9)

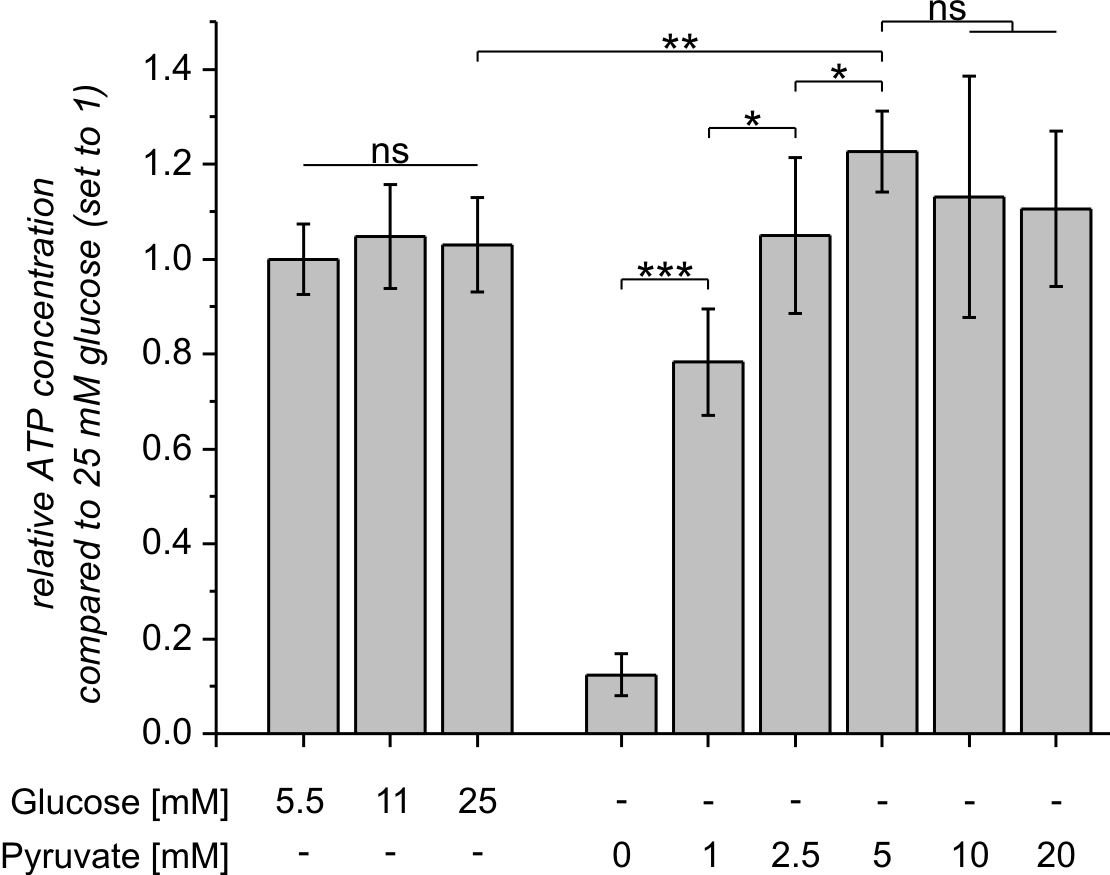

Supplement: Additional file 1: Figure S1. — Relative intracellular ATP concentration at different concentrations of glucose and pyruvate. U87 cells were seeded at a density of 5000 cells per well in 96 well microplates and received medium without a carbon source and without GlutaMAX and FBS for 20 h. Then, fresh medium was added containing different concentrations of glucose (5.5 mM, 11 mM and 25 mM), pyruvate (1 mM, 2.5 mM, 5 mM, 10 mM and 20 mM) or without any carbon source (0 mM). 24 h later the relative intracellular ATP concentration was determined by the CellTiterGlo Assay (for method description see Additional file 5: Methods). Results are represented as mean and standard deviation of 6 independently measured wells compared to the signal of 25 mM glucose set to 1. Statistical significance was determined by Student’s t-test with: *: p < 0.05; **: p < 0.005; ***: p < 0.0005; ns: not significant. (TIF 949 kb) [file 12986_2016_131_MOESM1_ESM.tif]

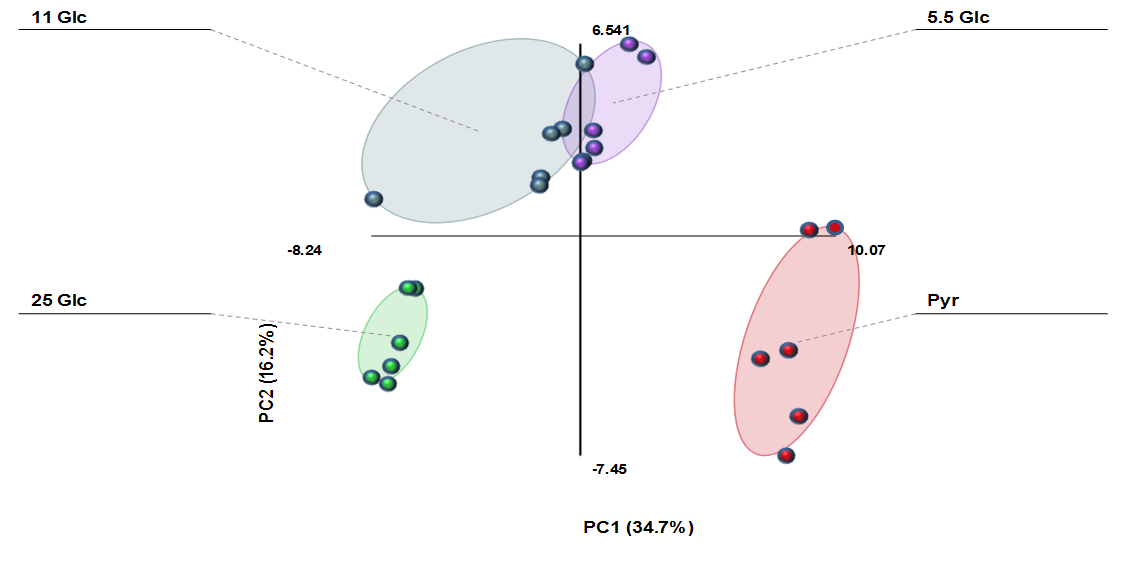

Supplement: Additional file 2: Figure S2. — Multivariate analysis of metabolite profiles. Principal component analysis (PCA) of metabolite profiles from U87 cells cultivated in the presence of 5.5 mM (5.5 Glc), 11 mM (11 Glc), 25 mM glucose (25 Glc) or 5 mM pyruvate in the absence of glucose (Pyr) for 24 h. 25 Glc and Pyr appear clearly separated from each other, whereas 11 Glc and 5.5 Glc have a similar profile separated from 25 Glc and Pyr. PC1 includes 34.7 % of variability (responsible for separation of Pyr), PC2 16.2 % (differences between the glucose treated cells). n = 6 for each condition, all identified metabolites were included (n = 106). (TIF 69 kb) [file 12986_2016_131_MOESM2_ESM.tif]

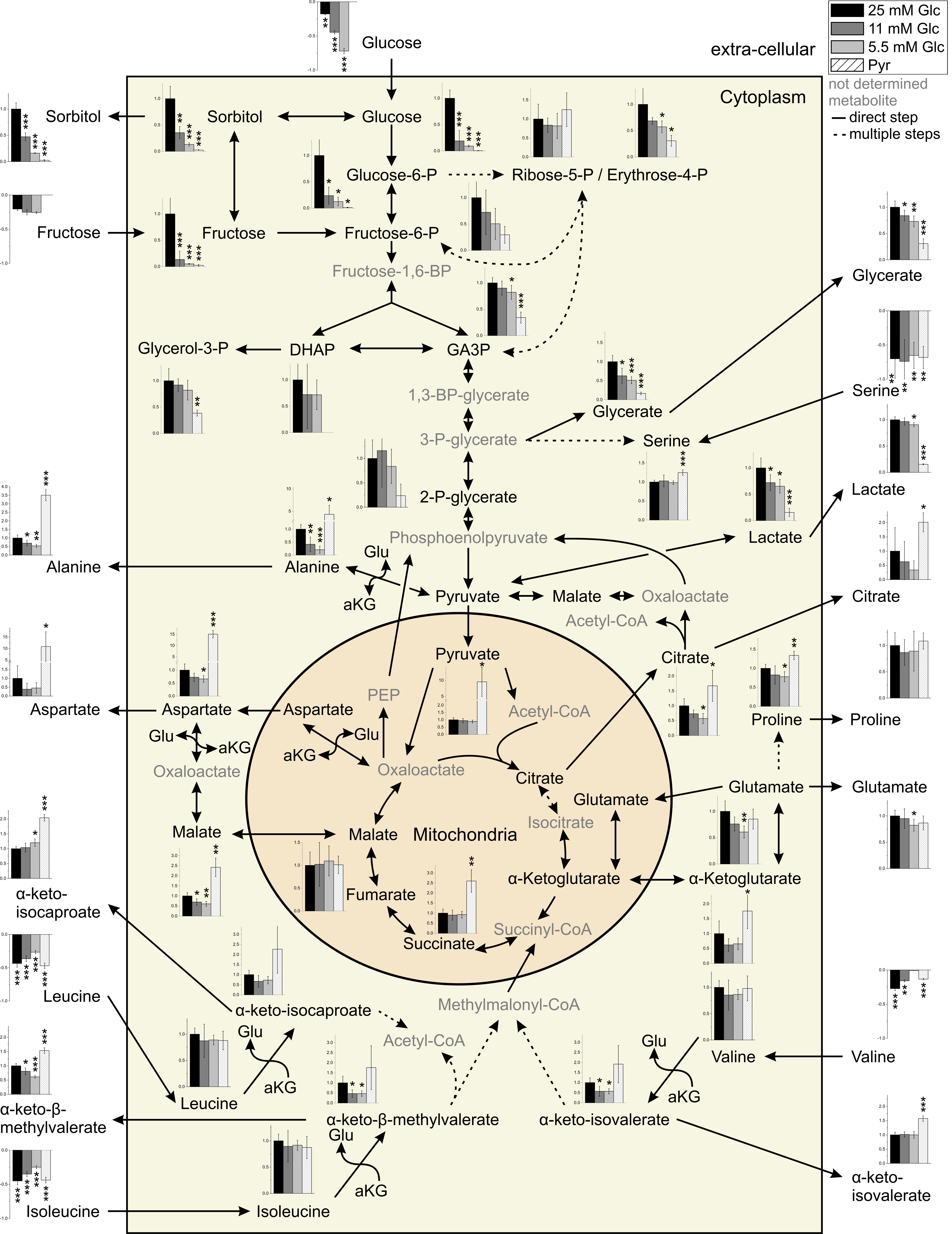

Supplement: Additional file 3: Figure S3. — Extended metabolic pathway of U87 cells profiling data. A combined metabolic pathway is presented, including glycolysis, the pentose-phosphate pathway, the TCA cycle and the branched chain amino acid degradation with the subcellular localization (mitochondrial, cytoplasm and extra cellular—methodically it can only be distinguished between intra- and extracellular metabolites). Intracellular metabolite abundances (Fig. 2) are shown as bar diagrams depicting the ratio between the abundance of a metabolite (the peak area) normalized to total protein (μg) compared to the abundance at 25 mM glucose (normalized to protein) set to 1. The consumption of extracellularly present metabolites was determined by the comparison of signals obtained from medium without cells to those after incubation with cells. Thus, a value of 0 indicates the same abundance of a metabolite in medium with and without cells (no consumption) and a value of -1 indicates that the metabolite is completely consumed. Abundances of metabolites released from the cells are expressed as the abundance of a metabolite in medium after incubation compared to its abundance in medium with 25 mM glucose set to 1. All experiments have been conducted in 6-tuplicate and statistical significance was determined by Student’s t-test with: *: p < 0.05; **: p < 0.005; ***: p < 0.0005. The statistical analysis for intracellular and released metabolites was performed by comparing the abundance of a metabolite in an experiment (11 mM or 5 mM glucose or 5 mM pyruvate) to the abundance in 25 mM glucose. For consumed metabolites, each metabolite’s abundance was compared to its abundance in medium without cells. Direct reactions are presented as straight lines and reactions involving several steps are presented as dotted lines. Metabolites which were not determined are labeled Grey. Abbreviations: GA3P: glyceraldehyde-3-phosphate; DHAP: dihydroxyacetone phosphate; Glu: glutamate; aKG: α-ketoglutarate; PEP: phosphoenolpyruvate. (JPG [file 12986_2016_131_MOESM3_ESM.jpg]

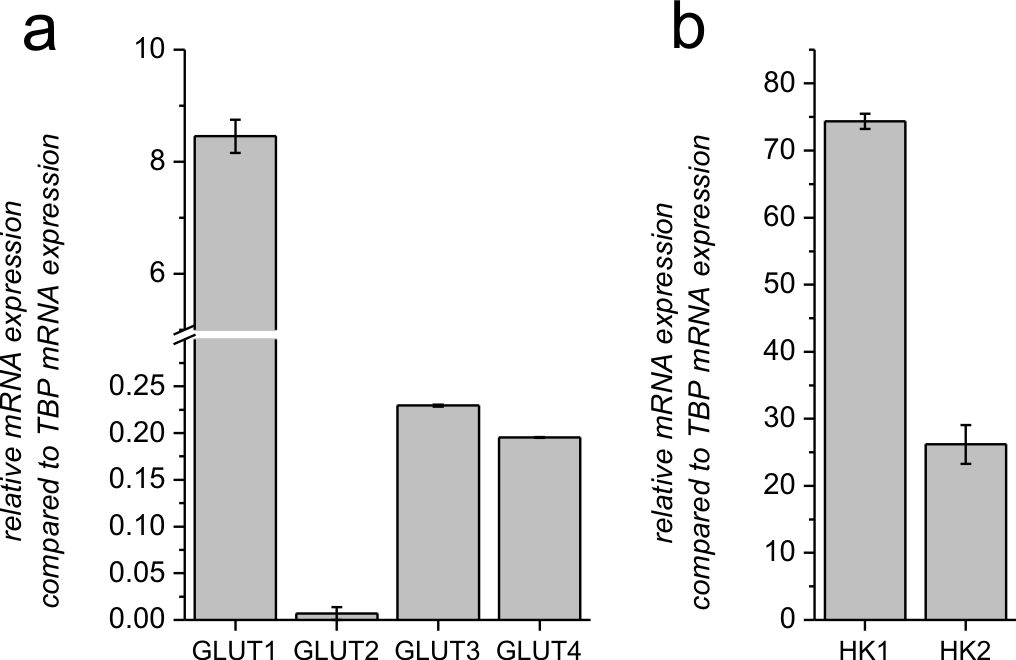

Supplement: Additional file 4: Figure S4. — mRNA expression of glycolytic genes in U87 cells. Expression of mRNA encoded by the genes GLUT1/2/3/4 (glucose transporters 1/2/3/4) (a) and HK1/2 (hexokinase 1/2) (b) in U87 cells as revealed by qRT-PCR (for method description see Additional file 5). The relative expression was determined using standard curves normalized to the expression of TATA box binding protein (TBP). (TIF 655 kb) [file 12986_2016_131_MOESM4_ESM.tif]
