# Supplementary material for: Metabolic response of glioblastoma cells associated with glucose withdrawal and pyruvate substitution as revealed by GC-MS
Source: Nutr Metab (Lond). 2016 Oct 18;13:70. doi: 10.1186/s12986-016-0131-9 (PMC5070012; doi:10.1186/s12986-016-0131-9)
Supplement: Additional file 5: — Methods. (DOCX 16 kb) [file 12986_2016_131_MOESM5_ESM.docx]

Supplementary methods

Cell based assays

Relative intracellular ATP levels were determined by the CellTiterGlo Assay (Promega, Mannheim, Germany) as described previously [1]. Briefly, 5000 U87 cells were seeded per well into a 96-well plate (µClear, Greiner Bio One, Frickenhausen, Germany) with 200 µl of full DMEM (4.5 g/L glucose, without pyruvate, supplemented with GlutaMAX, antibiotics – all from Gibco, Thermo scientific, Darmstadt, Germany – and 10 % FBS – Biochrom, Darmstadt, Germany). After 3 hours of incubation, media was exchanged with 100 µl of DMEM[0] (DMEM without glucose, pyruvate and phenol red, Gibco) and then cells were starved for 20 hours in order to exhaust energy resources. Then, cells received DMEM[0] supplemented with different concentrations of glucose and pyruvate. After 24 hours of incubation the CellTiterGlo Assay was performed and the resulting luminescence detected by a Mitras LB 940 Multimode Microplate ready (Berthold Technologies, Bad Wildbad, Germany). All assays were performed in 6-tuplicate.

Determination of mRNA expression by qRT-PCR

Determination of mRNA expression was performed as described before [2]. Briefly, for RNA isolation 10^6^ U87 cells were seeded into 10-mm cell cultured dishes (TPP, Trasadingen, Switzerland) with 10 ml of full DMEM. Cells received fresh media (full DMEM) after 24 hours and were cultivated for additional 24 hours. Then, total RNA was isolated using the RNeasy plus mini kit (Qiagen, Hilden, Germany) according to the manufactures instructions. For cDNA synthesis the imProm-II^TM^ Reverse Transcription System (Promega) was used according to the manufactures instructions, employing 500 ng RNA and random primers. qRT-PCR was carried out on a Rotor-Gene 3000 system (Qiagen) employing SYBR Green (Maxiam SYBR Greex/ROX qPCR Mastrer Mix, Thermo scientific) and the following primers: GLUT1_for: 5’-TGA TCG AGG AGT TCT ACA ACC AGA-3’; GLUT1_rev: CCGGGATGAAGATGATGCTC; GLUT2_for: ATTGCTCCAACCGCTCTCA; GLUT2_rev: CTGAAGGATGGCTCGCACACC; GLUT3_for: CTGGTTTATTGTGGCCGAACTCTT; GLUT3_rev: GCAGCATTCAGAAGCGTCCGT; GLUT4_for: GCTGTGTTCTCTGCGGTGCTT; GLUT4_rev: GCCTCCCCAGCCACGTCTCAT; HK1_for: GCAGATCTCTGAGACGCTGAA; HK1_rev: CTGTCATCGCAGGTGCTATTC; HK2_for: CCCTGCCACCAGACTAAACTA; HK2_rev: GCCCACAATGAGACCAATC; TBP_for: TTGACCTAAAGACCATTGCAC; TBP_rev: GCTCTGACTTTAGCACCTGTT. Copy numbers of the individual mRNAs were determined using linearized plasmid DNA containing the appropriate target sequence and normalized to the determined copy number of the reference gene TBP (for further information see [2]).

References

1. Gaunitz F, Heise K. HTS compatible assay for antioxidative agents using primary cultured hepatocytes. Assay Drug Dev Technol 2003;1:469–77. doi:10.1089/154065803322163786.

2. Letzien U, Oppermann H, Meixensberger J, Gaunitz F. The antineoplastic effect of carnosine is accompanied by induction of PDK4 and can be mimicked by l-histidine. Amino Acids 2014;46:1009–19. doi:10.1007/s00726-014-1664-8.
